# Influence of roundness errors of bearing components on rotational accuracy of cylindrical roller bearings

**DOI:** 10.1038/s41598-022-07718-y

**Published:** 2022-04-26

**Authors:** Yongjian Yu, Jishun Li, Yujun Xue

**Affiliations:** 1 School of Mechatronics Engineering, Henan University of Science and Technology, Luoyang, 471003 China; 2 Henan Key Laboratory for Machinery Design and Transmission System, Henan University of Science and Technology, Luoyang, 471003 China; 3 State Key Laboratory of Aviation Precision Bearings, Luoyang LYC Bearings Co., Ltd, Luoyang, 471039 China

**Keywords:** Aerospace engineering, Mechanical engineering

## Abstract

Understanding the influence of bearing component roundness errors and roller number on the rotational accuracy of rolling bearings is crucial in the design of high precision bearings. The rotational accuracy of an assembled bearing is dependent upon roller number and roundness errors of the bearing components. We propose a model for calculating the rotational accuracy of a cylindrical roller bearing; we experimentally verified the effectiveness of the model in predicting the radial run-out of the inner ring proposed in the previous paper in this series. We sought to define the key contributing factors to the rotational accuracy by studying both the influence of the coupling effect of the roller number and the influence of the roundness errors in the inner raceway, outer raceway, and rollers on the motion error. The model and results will help engineers choose reasonable manufacturing tolerances for bearing components to achieve the required rotational accuracy.

## Introduction

Rolling bearings are important mechanical parts commonly used in complex mechanisms, such as aircraft gas turbines, precision machine tools, disks, and gyroscopes. The rotational accuracy of an assembled bearing directly impacts the working accuracy of the mechanical equipment^1,2^. In manufacturing, the dynamic action and accuracy of the machine tool spindle will always introduce some degree of error into the bearing components. This roundness error is a critical factor in the motion error^3^ and must be studied to further improve the rotational accuracy of roller bearings.

Previous research on the rotational accuracy of rolling bearings mainly focused on the radial run-out. Bhateja et al.^4^ proposed a method for calculating the run-out of hollow roller bearings and studied the resultant components of the run-out from the geometric and dimensional errors in the rollers and raceways. Chen et al.^5,6^ proposed a method for calculating the radial run-out and static load distribution of cylindrical roller bearings and analyzed the effects of the roundness errors in the raceways and diameter differences of the rollers on the radial run-out and load distribution.

In the previous research in this series, Yu et al.^7,8^ proposed a method for calculating the radial run-out of the inner ring and analyzed the effects of the form error in the inner raceway and roller number on the radial run-out of cylindrical roller bearings. Yu et al.^9^, Li et al.^10^, and Liu et al.^11^ proposed a method for calculating the radial run-out of the outer ring considering roundness error of the outer raceway and investigated the influences of the roundness error, roller number, and radial clearance on the radial run-out in cylindrical roller bearings. Yu et al.^12^ proposed and experimentally verified a method for calculating the orbit of the outer ring center considering the geometric errors of the bearing components.

Researchers have also studied the influence of the component geometric error on the non-repetitive run-out (NRRO) and shaft axis orbit. Noguchi et al.^13–17^ developed a method for calculating the NRRO of ball bearings and theoretically investigated the effects of the ball number and element geometric error on the NRRO. Jang et al.^18^ analyzed the effect of viscoelastic damping on the NRRO of a ball bearing. Liu et al.^19^ and Tada et al.^20^ proposed prediction models for the NRRO of a ball bearing and analyzed the effect of the waviness of the inner groove, the outer groove, balls, and ball number on the NRRO. Ma et al.^21^ proposed a shaft center orbital method for spherical roller bearings and analyzed the influence of the roller diameter errors on the orbit of the shaft’s center. Okamoto et al.^22^ presented a calculation model for the ball bearing shaft axis orbit and investigated the influence of the form error, ball number, and ball diameter error on the shaft axis orbit.

Other researchers have investigated the influence of the waviness of the bearing components on the dynamic performance of roller bearings under different operating conditions. Wardle et al.^23,24^ and Ono et al.^25,26^ investigated the effect of element waviness on the dynamic performance of ball bearings. Talbot et al.^27^ investigated the influence of the macrogeometry of bearing components on the load intensities. Harsha et al.^28^, Wang et al.^29^, and Gunhee et al.^30^ analyzed the effect of the raceway and ball waviness on the dynamics of rigid rotor-bearing systems. Xu et al.^31,32^ and Kankar et al.^33^ analyzed the influence of waviness and localized defects on the dynamic performance of mechanisms. Shao et al.^34^ and Wang et al.^35^ investigated the effect of raceway localized defects on the bearing vibration. Tong et al.^36^ analyzed the influence of the form error on the tapered roller bearing performance. Petersen et al.^37^ investigated the influence of local defects and raceway roughness on the dynamics of a double row roller bearing. Podmasteriev^38^ analyzed the effect of the raceway geometric error on the probability of microcontacts in friction zones.

While there have been studies on the non-repetitive run-out and dynamic performance, there is relatively little research on the rotational accuracy of rolling bearings. Research on the rotational accuracy has mainly focused on the motion error of bearings derived from the combined action of the roller number and component roundness error in the process of rotation. The motion error of the bearing includes the run-out of the rotating ring in the horizontal and vertical directions of the radial plane.

In the current research on the rotational accuracy, many studies have investigated the effect of the component geometric error on the vertical run-out of the rotating ring. The vertical run-out of the rotating ring does not accurately reflect the run-out of the rotating ring in the radial plane, however, because it ignores the horizontal run-out of the rotating ring. We sought to identify the key contributing factors to the motion error of rolling bearings by studying both the influence of the coupling effect of the roller number and the influence of the component roundness errors on the run-out of the rotating ring in the radial plane. A motion error prediction model for cylindrical roller bearings was proposed in the previous paper of this series^39^ and is briefly described in “Prediction model for rotational accuracy of cylindrical roller bearings” section. The present study will experimentally verify the previously proposed model.

## Prediction model for rotational accuracy of cylindrical roller bearings

The rotary error of rolling bearings under the conditions of no load and a low speed determines the level of the rotational accuracy. As the rotary error decreases, the level of the rotational accuracy increases. The rotational accuracy of the rolling bearings is defined as the error between the position of the setting face and the ideal position of the rotating ring under the conditions of no load and a low speed.

No working load is applied to the bearing during measurements, but in order to maintain the operational stability of the bearing (full contact between the rolling elements and the raceway), it is necessary to apply a small measuring load to the bearing. This load must be small enough to not cause a visible elastic deformation between the bearing components. Low speeds prevent the impact between components and reduce bearing vibration, ensuring the measured motion error of the rolling bearing is only caused by roundness errors in the bearing components.

**Figure 1:**
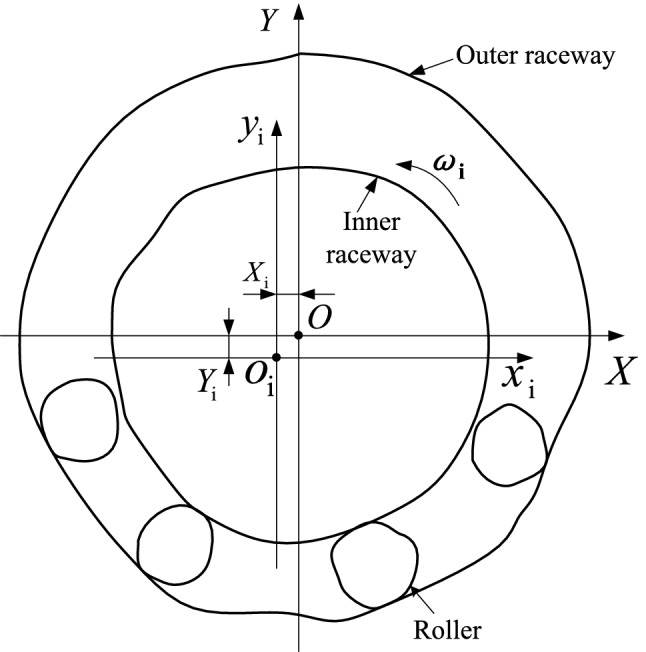
Geometric model of a bearing.

Figure 1 shows a diagram of a rolling bearing with the inner ring moving along the horizontal and vertical directions of the radial plane. Motion error occurs as the inner ring revolves around its axis because of the geometric errors in the raceways and rollers. In the pictured case, the inner raceway contacts the bottom part of rollers before the inner ring moves to an equilibrium position (*X*_i_, *Y*_i_). The coordinates of the inner ring’s center vary as it rotates. The previously developed prediction model was derived from a cylindrical roller bearing geometrical constraint model. The constraint model synthesizes both the geometric errors of the raceways and rollers and the change in real contact positions between raceways and rollers. The prediction model’s calculations iterate as described below:

1. The central coordinates of the bottom rollers contacting the outer raceway are calculated when the inner ring rotates one given step angle.
2. The inner ring moves in the radial plane and the contact statuses (contact, separation, and interference) between the inner raceway and rollers are determined for each given position.
3. The position of the inner ring in the radial plane is distinguished from other positions through the stable criterion based on the force equilibrium principle.
4. The distance between the centers of the inner ring and outer ring is calculated when the inner ring rotates a given angle.

Each time the inner ring rotates, the distance between the centers of the inner ring and outer ring is calculated by repeating the above calculations at different rotation angles. The difference between the maximum distance and the minimum distance derived from this process is the run-out variation of the inner ring, which reflects the run-out range of the inner ring.

## Verification of the prediction model

**Figure 2:**
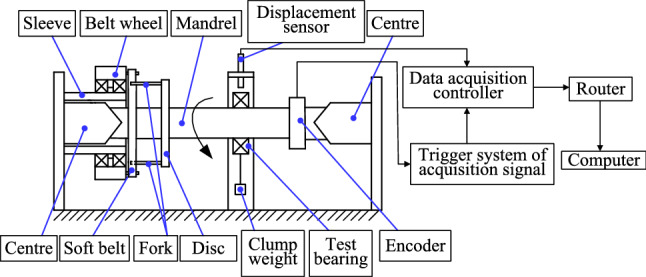
Schematic diagram to measure the radial run-out of the inner ring.

Figure 2 shows the basic composition of a device used to measure the rotational accuracy of a cylindrical roller bearing. Gauging principles and radial run-out methods are given in the international standard^40^. The disc with forks, the test bearing, and the encoder are fixed to the tapered mandrel. The mandrel is supported by a pair of coaxial centers so that it can only rotate along its axis. The measurement load is applied to the outer ring of the test bearing in the vertical direction in order to keep the test bearing stable. The large pulley is driven by the motor and small pulley. The mandrel is driven by the soft belt on the large pulley and the pair of forks on the disc. The inner ring of the test bearing turns with the mandrel.

When the inner ring rotates 0.7°, the acquisition signal trigger system sends a pulse signal to the data acquisition controller so that it collects the horizontal and vertical displacement data of the outer ring in the middle section once. The displacement of the outer ring corresponding to the number of rotation angles is obtained. The difference between the maximum and minimum displacement values is the radial run-out of the inner ring.

This method indirectly obtains the radial run-out of the inner ring by measuring the displacement of the outer ring. The inner ring is fixed on the spindle while the outer ring is static. The run-out of the inner ring relative to the outer ring is equivalent to the run-out of the outer ring relative to the inner ring.

**Figure 3:**
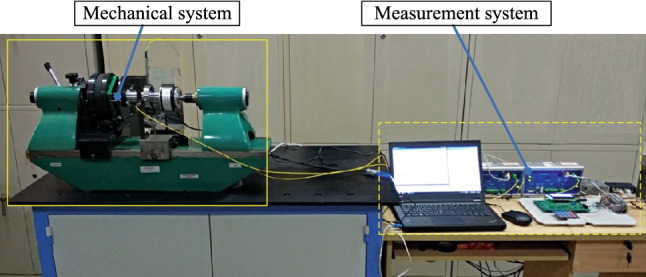
Test device to measure the radial run-out of the inner ring.

**Figure 4:**
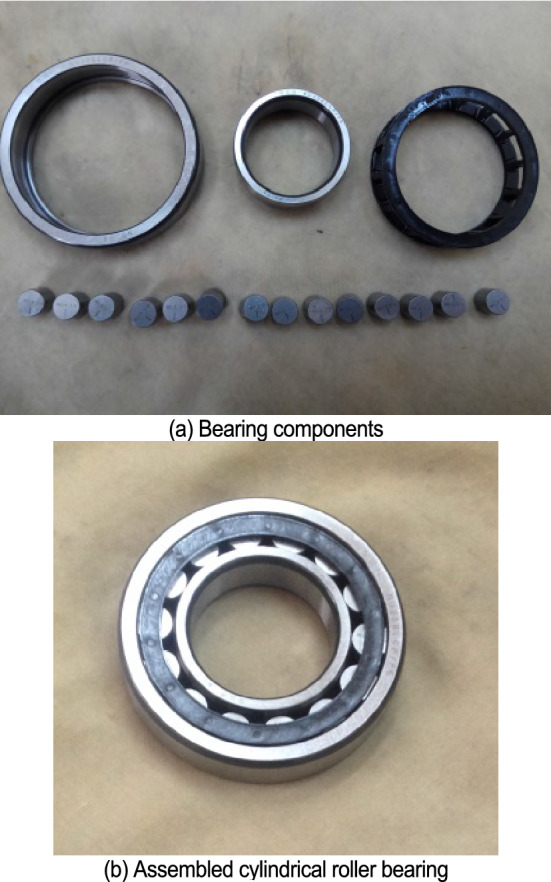
NU208 test bearings.

Figure 3 shows the test device used in this study to determine the run-out of the inner ring. Figure 4 shows the three sets of NU208 cylindrical roller bearings selected as test bearings.

The displacement of the inner ring depends on the relative position of the bearing components and so it is necessary to select an initial measurement state (relative positions of the inner ring, outer ring and roller) to be able to accurately compare modelled and experimental results. For every one rotation of the inner ring, 512 displacement measurements are collected.

In order to calculate the displacement of the inner ring with the prediction model, it is necessary to obtain the size and contour curve of the bearing components through tests. The contour curve of the bearing raceways is reconstructed through Fourier series where the parameters of the Fourier series are experimentally obtained. The profile data of the bearing raceways on the middle section is collected with a roundness instrument and the harmonic order, and its corresponding amplitude and phase angle are obtained through spectrum analysis of the data.

**Figure 5:**
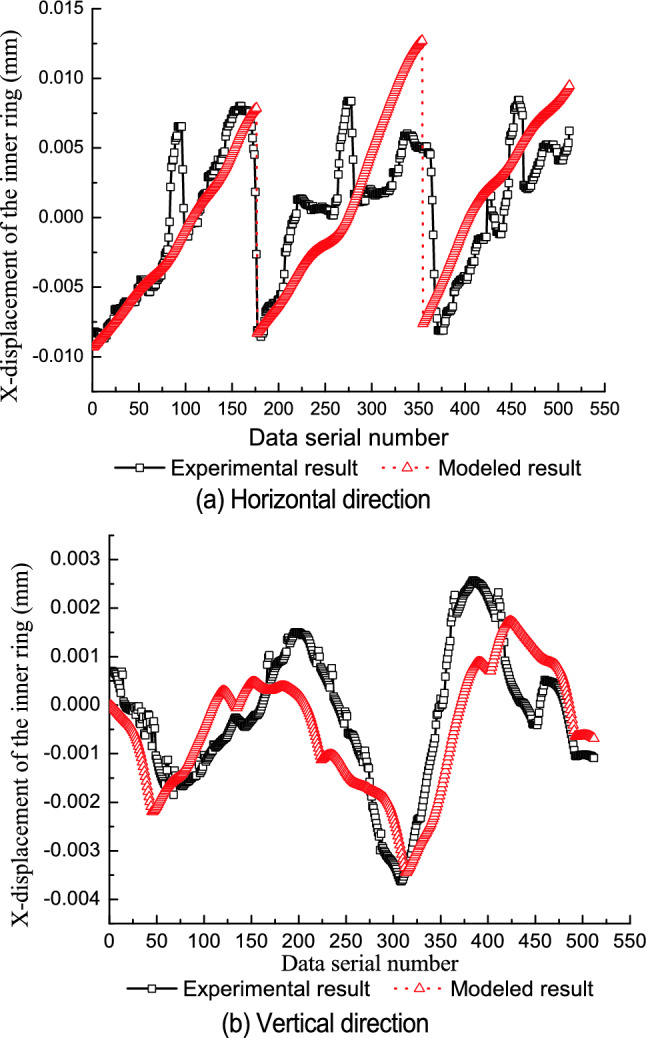
First test bearing comparison between the experimental results and modeled results.

**Figure 6:**
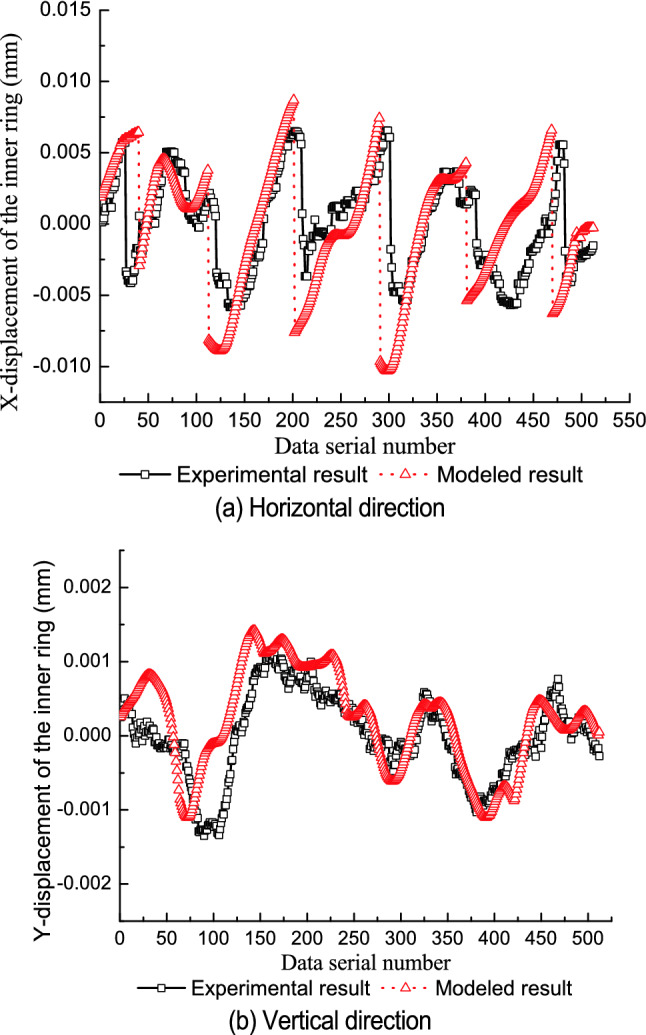
Second test bearing comparison between the experimental results and modeled results.

**Figure 7:**
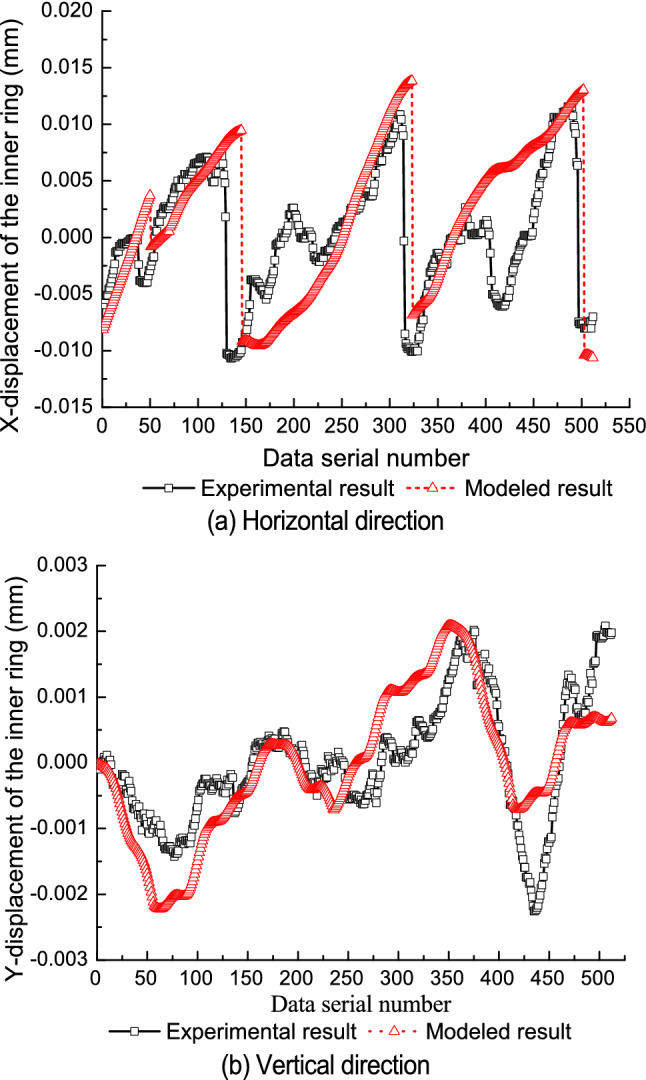
Third test bearing comparison between the experimental results and modeled results.

Table 1 shows the parameters of the test bearings. The relative position of the components is consistent with the initial test state of the test bearing. The roundness error of the rollers is ignored. For every one rotation of the inner ring, 512 displacement points are collected by the predication model. Figures 5, 6 and 7 compare the modeled results with the experimental results for each of the three test bearings.

**Table 1:** Part diameter of test bearings.

Test bearing	Inner raceway (mm)	Outer raceway (mm)	Rollers (mm)
No. 1	49.489	71.542	10.998–10.999
No. 2	49.492	71.540	10.997–10.999
No. 3	49.499	71.545	10.997–11.000

**Figure 8:**
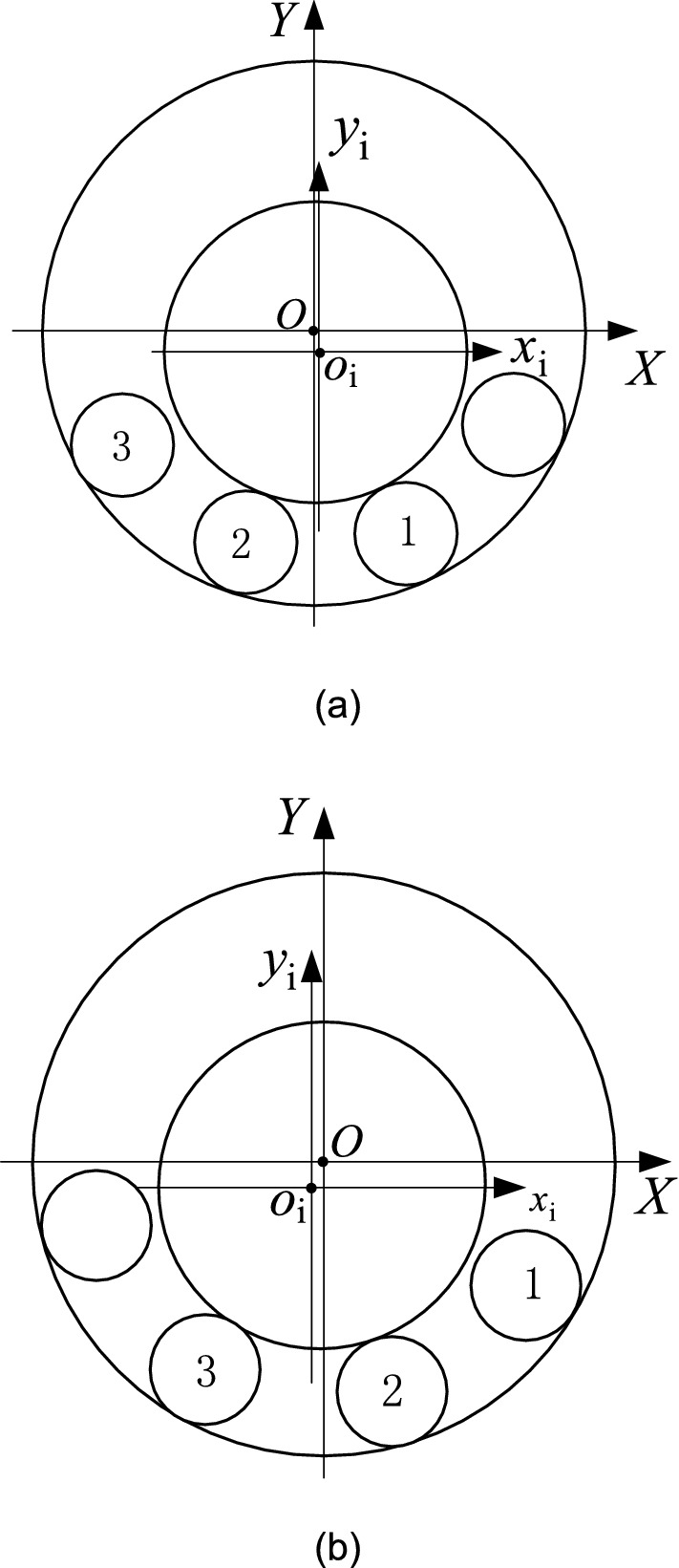
Contact status of inner ring with inner raceway for an ellipse.

The model’s predicted values are consistent with the experimental results in the vertical direction and in the horizontal direction. From Figs. 5a, 6a and 7a, it can be seen that jump phenomena exists in the X displacement of the inner ring. The reasons for this phenomenon are as follows. When the amplitude of roundness error in the inner raceway is relatively small, only the No.1 and No.2 rollers contact with the raceways under geometric constraints, as shown in Fig. 8a. When the No.2 roller rolls from the third quadrant to the fourth quadrant, the No.3 roller contacts with the raceways and the No.1 roller is separate from the inner raceway. At this point, the rollers which contact with the raceways change from the No.1 and No.2 roller to the No.2 and the No.3 roller, and the center position of the inner ring varies from the fourth quadrant to the third quadrant, as shown in Fig. 8b. Therefore, the X coordinate of the inner ring changes from the positive value to the negative value, thus resulting in a jump in the X displacement of the inner ring.

Differences between the prediction results and the experimental results are most likely due to.

1. The initial state of the bearing depends on the initial state and relative position of the inner ring, outer ring, and all rollers. The initial state of the bearing directly determines the interaction contour shape among the bearing components and affects the displacement of the inner ring. It is difficult to ensure that the initial state of the bearing components is completely consistent with the state defined in the theoretical prediction.
2. The purpose of the test is to measure the displacement of the outer ring (equivalent to the displacement of the inner ring) caused by the geometric error of the bearing components. Rotation error of the spindle will also cause some displacement of the outer ring, however, because the spindle rotates synchronously with the inner ring. The actual measured displacement of the outer ring may include this spindle error while the predicted displacement does not.
3. It is difficult to apply the measured force to the outer ring along the vertical direction, which may deviate the test state of the test bearing from the theoretical state and cause differences between the predicted results and the experimental results.
4. The axial geometry error and shape error of all rollers are not considered in the predicted results, but may affect the measured results.

## Results and discussion

Table 2 shows the main parameters of the NU208 type cylindrical roller bearings used in this study. The influence of the roller number and roundness errors in the inner raceway, the outer raceway, and the rollers on the run-out variation of the inner ring is analyzed. The coupling effect of the roller number and component roundness error on the run-out variation of the inner ring is examined in this section.

**Table 2:** Main parameters of cylindrical roller bearing.

Parameters	Value
Inner raceway diameter (mm)	49.499
Outer raceway diameter (mm)	71.545
Roller diameter (mm)	11
Number of rollers	14
Radial internal clearance (mm)	0.041

### Effect of roundness error in the inner raceway

#### Effect of the order of roundness error

**Figure 9:**
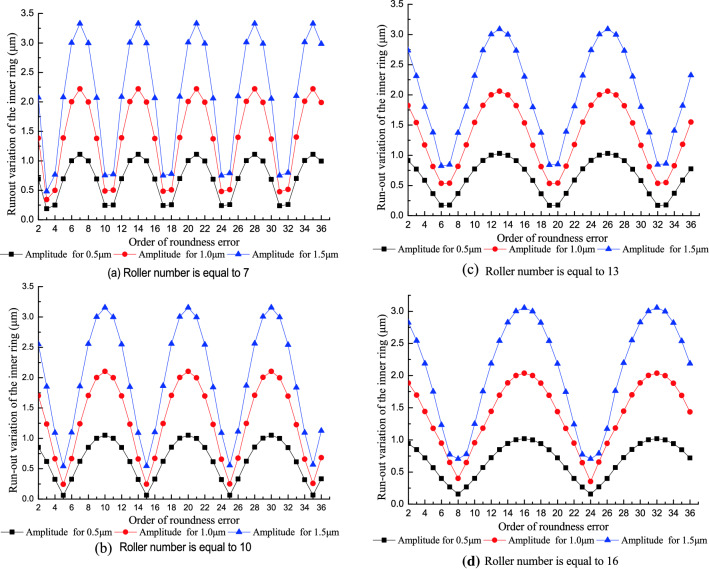
Effect of the order of the roundness error in the inner raceway on the run-out variation of the inner ring.

Figure 9 shows the effect of the order of roundness error in the inner raceway on the run-out variation of the inner ring at different roller numbers. The predicted results (Fig. 9) indicated the run-out variation of the inner ring approximates a sinusoidal curve with the increasing order of the roundness error and period equals to *Z* (roller number). The increase in the run-out variation of the inner ring is proportional with the increase in the amplitude of the roundness error in the inner raceway. The effect of the order of the roundness error on the run-out variation of the inner ring changes with the roller number.

When the order of roundness error is equal to (2*n* − 1)*Z*/2 (where n is a natural number and *Z* is an even number) or (*Z* ± 1)/2 + (*n* − 1)*Z* (where *Z* is an odd number), the run-out variation of the inner ring reaches its minimum. When the order of roundness error is equal to *nZ*, the run-out variation of the inner ring reaches its maximum. This trend occurs because the model assumes the inner ring rotates. When the order of roundness error equals (2*n* − 1)*Z*/2, each time a roller undulates to a peak or valley, the adjacent roller also undulates to the opposite peak or valley and causes a minimum run-out variation of the inner ring. When the order of the roundness error equals *nZ*, the adjacent rollers in the contact zone simultaneously undulate to the same peak or valley and cause a maximum run-out variation of the inner ring. In order to effectively improve the rotational accuracy of the assembled bearing, the harmonic components of the inner raceway with integral multiple orders of the roller number should be controlled in the component machining process.

#### Effect of the amplitude of roundness error

**Figure 10:**
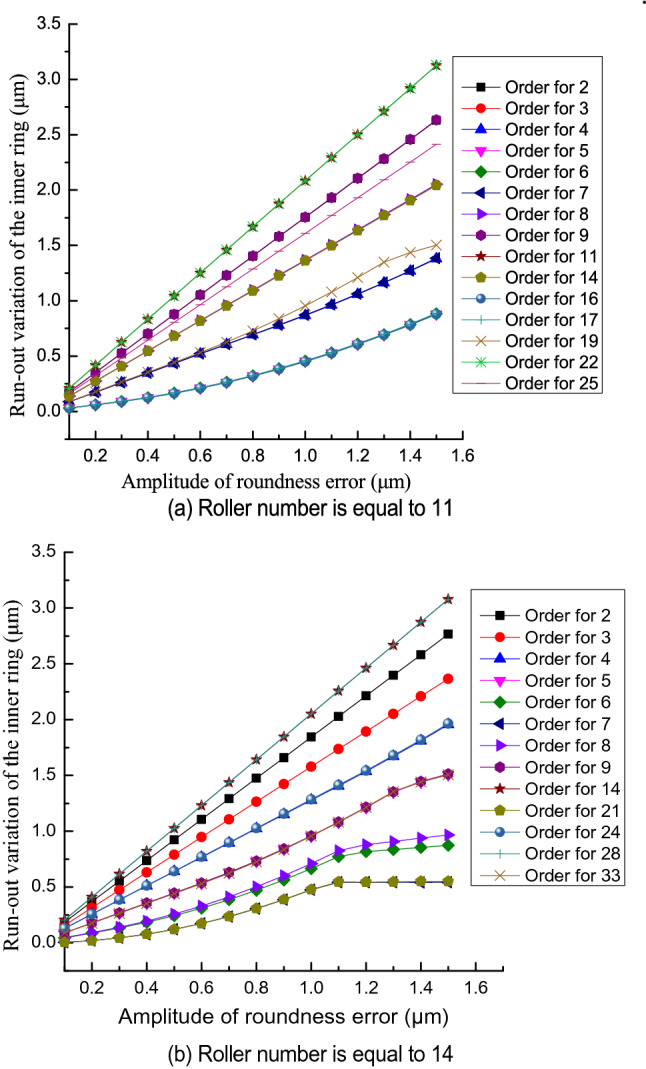
Effect of the amplitude of the roundness error in the inner raceway on the run-out variation of the inner ring.

Figure 10 shows the relationship between the run-out variation of the inner ring and the amplitude of roundness error in the inner raceway. The run-out variation of the inner ring increases as the amplitude of the roundness error increases. The significant rise in the run-out variation of the inner ring occurs because the increase in the amplitude of the roundness error increases the height between the peak and valley of the undulation and so increases the inner ring maximum run-out distance and decreases the minimum run-out distance.

The effect of the amplitude of the roundness error on the run-out variation of the inner ring changes with the roller number and the order of the roundness error. When the order of the roundness error is equal to (2*n* − 1)*Z*/2 (where n is a natural number and *Z* is an even number) or to (*Z* ± 1)/2 + (*n* − 1)*Z* (where *Z* is an odd number), the amplitude of the roundness error in the inner raceway has less influence on the run-out variation of the inner ring. When the order of the roundness error is equal to *nZ*, the amplitude of the roundness error in the inner raceway has a significant effect on the run-out variation of the inner ring.

### Effect of roundness error in the outer raceway

#### Effect of the order of roundness error

**Figure 11:**
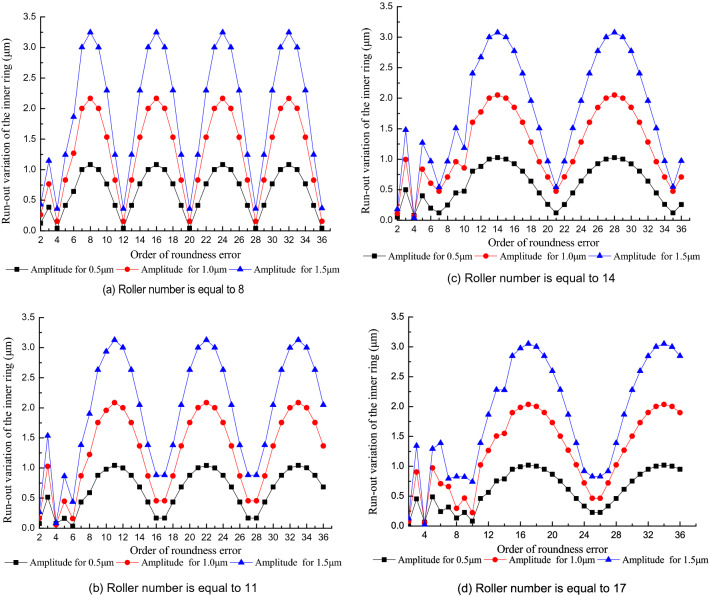
Effect of the order of roundness error in the outer raceway on the run-out variation of the inner ring.

Figure 11 shows the influence of the order of the roundness error in the outer raceway on the run-out variation of the inner ring at different roller numbers. Figure 11 indicates that when the order of the roundness error of the outer raceway is more than half the roller number, the run-out variation of the inner ring approximates a sinusoidal curve with increasing order of roundness error and period equals to *Z*. The increase in the run-out variation of the inner ring is proportional with the increase of the amplitude of the roundness error in the outer raceway.

Figure 11 also indicates that the run-out variation of the inner ring reaches its minimum when the order of roundness error is equal to (2*n* + 1)Z/2 (where *Z* is an even number) or to (Z ± 1)/2 + (2*n* − 1)*Z* (where *Z* is an odd number). When the order of the roundness error is equal to *nZ*, the run-out variation of the inner ring reaches its maximum. When the order of roundness error is less than half the roller number the run-out variation of the inner ring has a strongly nonlinear variation as the roller number increases. This trend occurs because the outer ring does not rotate and the profile of the outer raceway in the contact zone of the bearing is less than one periodic profile of the outer raceway. The run-out variation of the inner ring results from part of one periodic profile.

When the roller number increases the run-out variation of the inner ring shows a strongly nonlinear variation, not a periodic variation. In order to effectively improve the rotational accuracy of an assembled bearing the harmonic components of the outer raceway with integral multiple orders of the roller number should be controlled in the component machining process.

#### Effect of the amplitude of roundness error

**Figure 12:**
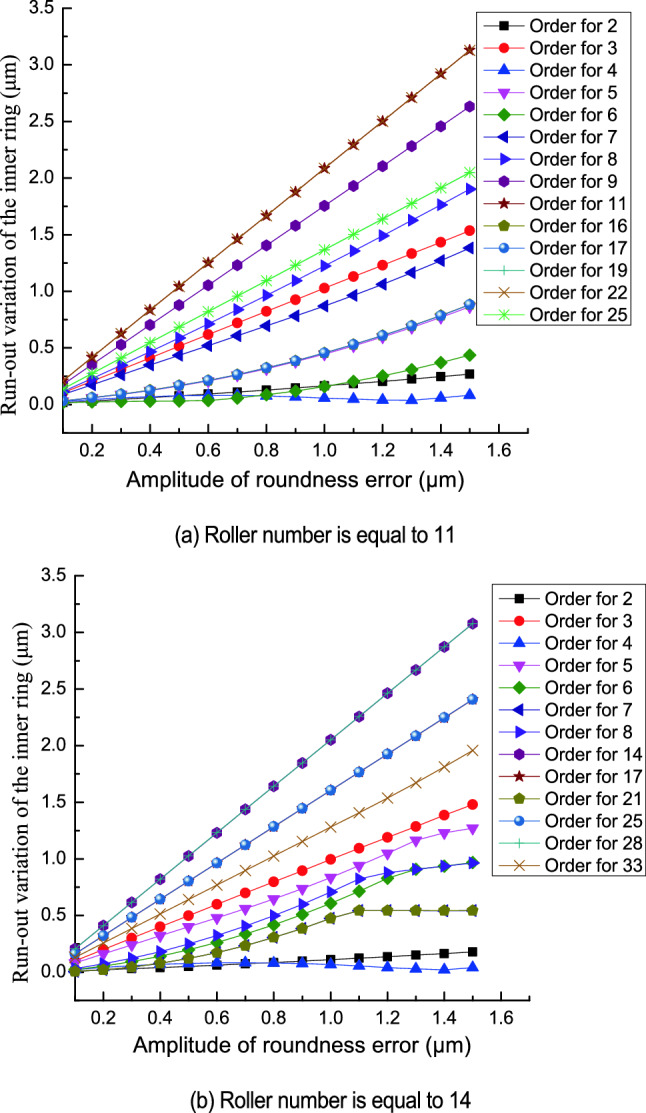
Effect of the amplitude of roundness error in the outer raceway on the run-out variation of the inner ring.

Figure 12 shows the relationship between the run-out variation of the inner ring and the amplitude of roundness error in the outer raceway. The run-out variation of the inner ring increases linearly as the amplitude of roundness error increases. The effect of the amplitude of the roundness error on the run-out variation of the inner ring changes with the roller number and order of the roundness error. When the order of roundness error is less than half of the roller number, and the order of roundness error is equal to 2 or 4 the amplitude of roundness error has less influence on the run-out variation of the inner ring. When the order of roundness error is more than half of the roller number and is equal to (2*n* − 1)*Z*/2 (where n is a natural number and *Z* is an even number) or to (*Z* ± 1)/2 + (2*n* − 1)*Z* (where *Z* is an odd number) the amplitude of roundness error in the outer raceway has less influence on the run-out variation of the inner ring. When the order of roundness error is equal to *nZ* the amplitude of roundness error in the outer raceway has a significant effect on the run-out variation of the inner ring.

### Effect of roundness error in the rollers

#### Effect of the order of roundness error

**Figure 13:**
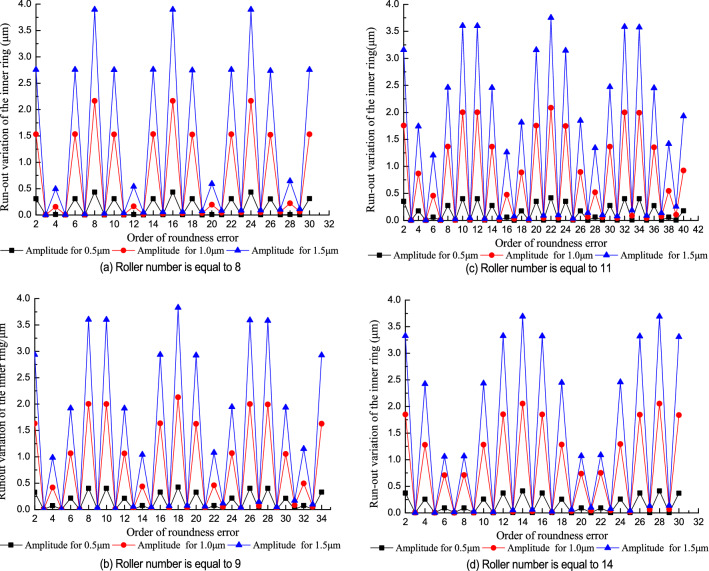
Effect of the order of roundness error in the rollers on the run-out variation of the inner ring.

Figure 13 shows the effect of the order of roundness error in the rollers on the run-out variation of the inner ring. In order to analyze the coupling effect of the roller number and the order of roundness error in rollers on the run-out variation of the inner ring the effect of the order of roundness error at different roller numbers is given. Run-out variation of the inner ring fluctuates periodically with the increase of the order of roundness error, and period depends on the parity of the roller number. When the roller number is even the period is equal to *Z*. When the roller number is odd the period is equal to 2*Z*.

The run-out variation of the inner ring reaches its maximum when the order of roundness error is odd. When the order of roundness error is even the run-out variation of the inner ring reaches its minimum and for even the amplitude of roundness error, the run-out variation of the inner ring is less than 0.1 μm. The increase in the run-out variation of the inner ring is proportional with the increase of the amplitude of roundness error in the rollers. The effect of even orders of roundness error in the rollers on the run-out variation of the inner ring is significant, and the effect of odd orders of roundness error in the rollers on the run-out variation of the inner ring is negligible.

#### Effect of the amplitude of roundness error

**Figure 14:**
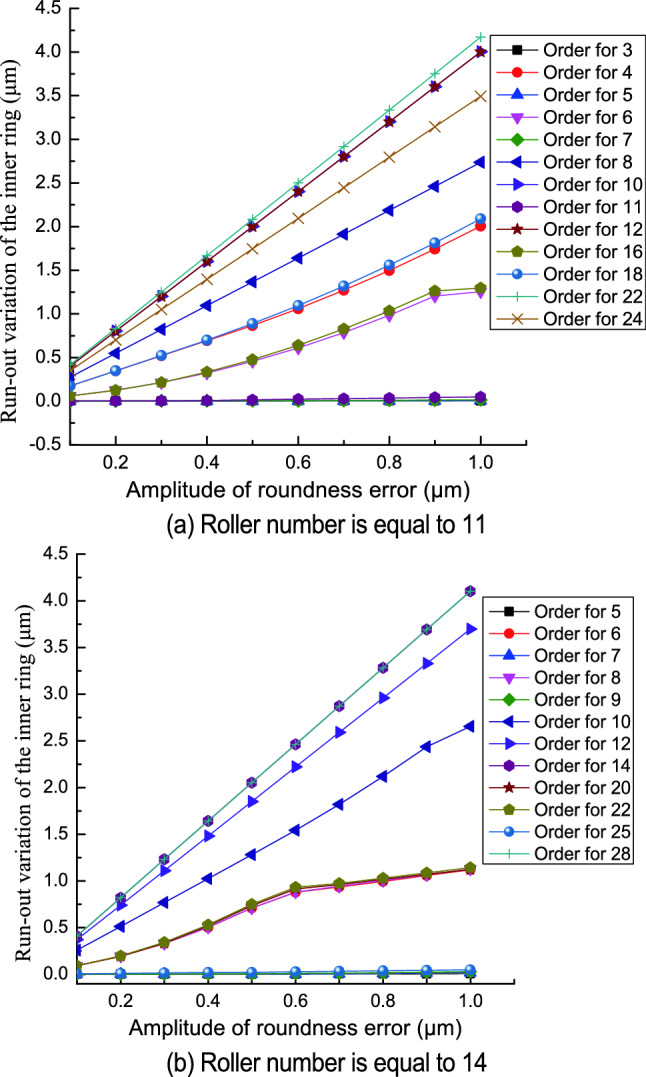
Effect of the amplitude of roundness error in the rollers on the run-out variation of the inner ring.

Figure 14 shows the effect of the amplitude of the roundness error in the rollers on the run-out variation of the inner ring. The run-out variation of the inner ring increases linearly as the amplitude of the roundness error in the rollers increases, and the effect of the amplitude of the roundness error on the run-out variation of the inner ring changes with the roller number and order of the roundness error. When the order of the roundness error is even, the run-out variation of the inner ring sharply increases with the amplitude of the roundness error. When the order of the roundness error is even and equals (*Z* − 1)/2 ± 1 (where *Z* is an odd number) or *Z*/2 ± 1 (where Z/2 is an odd number), the amplitude of the roundness error in the rollers has less influence on the run-out variation of the inner ring. When the order of the roundness error is even and equals *nZ* (where *nZ* is an even number), the amplitude of the roundness error in the rollers has a significant effect on the run-out variation of the inner ring. When the order of the roundness error is odd, the run-out variation of the inner ring slightly increases as the amplitude of the roundness error increases but is always less than 0.07 μm. The effect of the amplitude of the roundness error with even orders on the run-out variation of the inner ring is significant, and the effect of the amplitude of the roundness error with odd orders on the run-out variation of the inner ring is negligible.

### Effect of the roller number

#### Effect of the roller number with roundness error in the inner raceway

**Figure 15:**
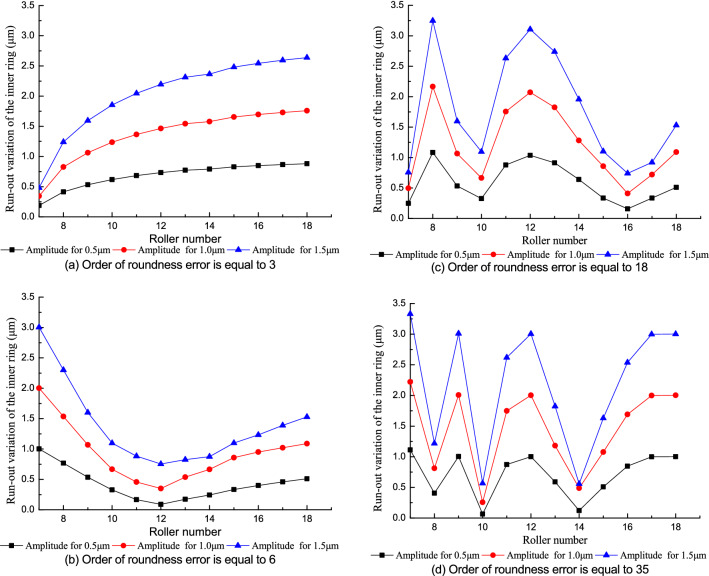
Effect of the roller number on the run-out variation of the inner ring.

Figure 15 shows the influence of the roller number on the run-out variation of the inner ring when there is a roundness error in the inner raceway. The effect of the roller number varies with the order of the roundness error in the inner raceway. When the order of the roundness error is large, the run-out variation of the inner ring shows a significant nonlinear tread with the increasing roller number because of the coupling effect of the roller number and the order of the roundness error makes the extreme point of run-out variation of the inner ring increase significantly with the increasing order of the roundness error. Increasing the roller number does not always result in a decrease of the run-out variation in the inner ring when there is a roundness error in the inner raceway.

#### Effect of the roller number with roundness error in the outer raceway

**Figure 16:**
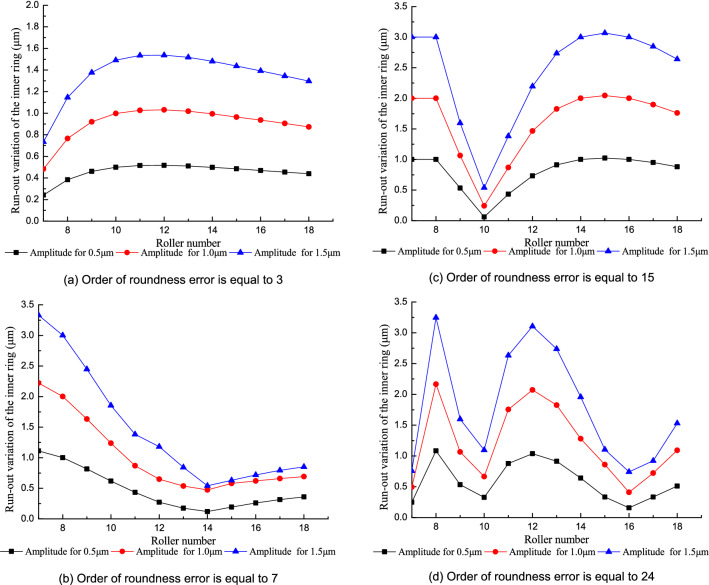
Effect of the roller number on the run-out variation of the inner ring.

Figure 16 shows the influence of the roller number on the run-out variation of the inner ring when there is a roundness error in the outer raceway. The effect of the roller number on the run-out variation of the inner ring changes with the order of the roundness error in the outer raceway. When the order of the roundness error is large, the run-out variation of the inner ring fluctuates more as the roller number increases because of the coupling effect of the roller number and the order of the roundness error in the outer raceway becomes strong with the increase of the order of the roundness error. Increasing the roller number does not always decrease the run-out variation in the inner ring when there is a roundness error in the outer raceway.

## Conclusions

1. The amplitude and order of the roundness error in the inner and outer raceway has a significant impact on the motion error of the cylindrical roller bearings. When the order of the roundness error in the inner raceway is equal to (2*n* − 1)*Z*/2 (where n is a natural number and Z is an even number) or to (*Z* ± 1)/2 + (*n* − 1)*Z* (where *Z* is an odd number) or the order of roundness error in the outer raceway is equal to (2*n* + 1)*Z*/2 (where *Z* is an even number) or to (*Z* ± 1)/2 + (2*n* − 1)Z (where *Z* is an odd number), the roundness error in the raceways significantly reduces the motion error of the bearing and improves the rotational accuracy of the assembled bearing. When the order of the roundness error in the inner or outer raceway is equal to *nZ*, the roundness error in the raceways significantly increases the motion error of the bearing and the rotational accuracy of the assembled bearing decreases significantly. It is crucial to avoid producing harmonic components where the order is an integer multiple of the roller number in the machining process of the inner and outer raceways to improve the rotational accuracy of cylindrical roller bearings.
2. The effect of the even-order roundness error in the rollers on the motion error of cylindrical roller bearings is significant, while the odd-order roundness error in the rollers does not affect the motion error. Even-order harmonic components in the rollers should be strictly controlled in order to improve the rotational accuracy of the assembled bearing.
3. Increasing the roller number does not always decrease motion error of the cylindrical roller bearings because of the coupling effect of the roller number and roundness error of the bearing components. The roller number should be matched with the harmonic order of the bearing components to effectively improve the rotational accuracy of the assembled bearing.

## List of symbols

*n*: Positive integer
*Z*: Roller number

## References

[1] Takabi J, Khonsari MM (2015). On the dynamic performance of roller bearings operating under low rotational speeds with consideration of surface roughness. Tribol. Int..

[2] Sayles RS, Poon SY (1981). Surface topography and rolling element vibration. Precis. Eng..

[3] Jiang SY, Zheng SF (2010). Dynamic design of a high-speed motorized spindle-bearing system. J. Mech. Des..

[4] Bhateja CP, Pine RD (1981). The rotational accuracy characteristics of the preloaded hollow roller. J. Lubr. Technol..

[5] Chen GC, Wang BK, Mao FH (2013). Effects of raceway roundness and roller diameter errors on clearance and runout of a cylindrical roller bearing. Proc. Inst. Mech. Eng. Part J J. Eng. Tribol..

[6] Chen GC, Mao FH, Wang BK (2012). Effects of off-sized cylindrical rollers on the static load distribution in a cylinder roller bearing. Proc. Inst. Mech. Eng. Part J J. Eng. Tribol..

[7] Yu YJ, Li JS, Chen GD (2017). Rotational accuracy of the cylindrical roller bearing based on inner raceway profile. Chin. J. Aerosp. Power.

[8] Yu, Y. J., Chen, G. D., Li, J. S., *et al.* Research on rotational accuracy of cylindrical roller bearings. In *10th CIRP Conference on Intelligent Computation in Manufacturing Engineering, Naples, Italy* (2016).

[9] Yu YJ, Li JS, Chen GD (2016). Numerical calculation and experimental research of rotational accuracy on cylindrical roller bearing. Chin. J. Mech. Eng..

[10] Li, J. S., Yu, Y. J., Xue, Y. J. Forecast for radial runout of outer ring in cylindrical roller bearing. In *10th CIRP Conference on Intelligent Computation in Manufacturing Engineering, Naples, Italy* (2016).

[11] Liu YG, Li JS, Shi WX, Jia XZ (2011). An Algorithm to prediction the radial runout of cylindrical roller bearings. Appl. Mech. Mater..

[12] Yu YJ, Chen GD, Li JS (2017). A Method to predict the radial runout of outer ring in cylindrical roller bearings. Adv. Mech. Eng..

[13] Noguchi S, Ono K (2004). Reduction of NRRO in ball bearings for HDD spindle motors. Precis. Eng..

[14] Noguchi S, Kentaro H, Hiroyuki K (2005). The Influence of location of balls and ball diameter difference in rolling bearings on the non-repetitive runout (NRRO) of retainer revolution. Precis. Eng..

[15] Noguchi S, Hiruma K (2003). Theoretical analysis of the NRRO of the components of retainer rotation in consideration of diameter difference and disposition of balls in a ball bearing. Jpn. J. Tribol..

[16] Noguchi S, Obinata S, Saito Y (2005). Influence of the unequal orbital intervals of balls on the NRRO of rotational frequency of the cage in a ball bearing. Jpn. J. Tribol..

[17] Noguchi S, Tanaka K (1999). Theoretical analysis of a ball bearing used in HDD spindle motors for reduction of NRRO. IEEE Trans. Magn..

[18] Jang GH, Kim DK, Han JH (2001). Characterization of NRRO in a HDD spindle system due to ball bearing excitation. IEEE Trans. Magn..

[19] Liu J, Hong J, Yang ZH (2011). Running accuracy of high speed precision angular contact ball bearings. J. Xi’an Jiaotong Univ..

[20] Tada S (2002). Three-dimensional analysis of non-repeatable runout( NRRO) in ball bearing. KOYO Eng. J. Engl. Ed..

[21] Ma FB, Ji P, Li ZM (2015). Influences of off-sized rollers on mechanical performance of spherical roller bearings. J. Multibody Dyn..

[22] Okamoto J, Ohmori T (2001). Study on run-out of ball bearings—relation between unroundness of race and locus of shaft in rotation. Jpn. J. Tribol..

[23] Wardle FP (1988). Vibration forces produced by waviness of the rolling surfaces of thrust loaded ball bearings Part 1: Theory. Proc. Inst. Mech. Eng. Part C J. Mech. Eng. Sci..

[24] Wardle FP (1988). Vibration forces produced by waviness of the rolling surfaces of thrust loaded ball bearings Part 2: experimental validation. Proc. Inst. Mech. Eng. Part C J. Mech. Eng. Sci..

[25] Ono K, Takahasi K (1996). Theoretical analysis of shaft vibration supported by a ball bearing with small sinusoidal waviness. IEEE Trans. Magn..

[26] Ono K, Okada Y (1998). Analysis of ball bearing vibrations caused by outer race waviness. J. Vib. Acoust..

[27] Talbot D, Li S, Kahraman A (2013). Prediction of mechanical power loss of planet gear roller bearings under combined radial and moment loading. J. Mech. Des..

[28] Harsha SP, Kankar PK (2004). Stability analysis of a rotor bearing system due to surface waviness and number of balls. Int. J. Mech. Sci..

[29] Wang LQ, Cui L, Zheng DZ (2008). Nonlinear dynamics behaviors of a rotor roller bearing system with radial clearances and waviness considered. Chin. J. Aeronaut..

[30] Jang G, Jeong S-W (2004). Vibration analysis of a rotating system due to the effect of ball bearing waviness. J. Sound Vib..

[31] Xu L, Li Y (2015). Modeling of a deep-groove ball bearing with waviness defects in planar multibody system. Multibody Syst. Dyn..

[32] Xu LX, Yang YH (2015). Modeling a non-ideal rolling ball bearing joint with localized defects in planar multibody systems. Multibody Syst. Dyn..

[33] Kankar PK, Sharma Satish C, Harsha SP (2012). Vibration based performance prediction of ball bearings caused by localized defects. Nonlinear Dyn..

[34] Shao Y, Liu J, Ye J (2014). A New method to model a localized surface defect in a cylindrical roller-bearing dynamic simulation. Proc. Inst. Mech. Eng. Part J J. Eng. Tribol..

[35] Wang F, Jing M, Yi J (2015). Dynamic modelling for vibration analysis of a cylindrical roller bearing due to localized defects on raceways. Proc. Inst. Mech. Eng. Part K J. Multi-body Dyn..

[36] Tong V-C, Hong S-W (2015). Characteristics of tapered roller bearing with geometric error. Int. J. Precis. Eng. Manuf..

[37] Petersen D, Howard C, Sawalhi N (2015). Analysis of bearing stiffness variations, contact forces and vibrations in radially loaded double row rolling element bearings with raceway defects. Mech. Syst. Signal Process..

[38] Podmasteriev KV (2000). Effect of raceway form errors on probability of microcontacts in bearing. J. Frict. Wear.

[39] Yu YJ, Chen GD, Li JS (2017). Prediction method for the radial runout of inner ring in cylindrical roller bearings. Math. Probl. Eng..

[40] ISO 1132–2—2001. *Rolling Bearings—Tolerances Part 2: Measuring and Gauging Principles and Methods*, (2001).

